# Is Left Ventricular Assist Device Deactivation Ethically Acceptable? A Study on the Euthanasia Debate

**DOI:** 10.1007/s10730-020-09408-6

**Published:** 2020-04-06

**Authors:** Sara Roggi, Mario Picozzi

**Affiliations:** 1 Center for Clinical Ethics (CREC), Doctoral School in Clinical and Experimental Medicine and Medical Humanities, Biotechnologies and Life Sciences Department, Insubria University, Via Ottorino Rossi 9, 21100 Varese, Italy; 2 Centre de Recherche sur le Liens Sociaux (CERLIS), Doctoral School 180 in Human et Social Sciences: Cultures, Individuals and Societies, Paris Descartes University, Galerie Gerson, 1st Floor, 54, Rue Saint Jacques, 75005 Paris, France; 3 Center for Clinical Ethics, Biotechnologies and Life Sciences Department, Insubria University, Via Ottorino Rossi 9, 21100 Varese, Italy

**Keywords:** Device, Left ventricular assist device, Deactivation, Euthanasia, Proportionality

## Abstract

In the last decades, new technologies have improved the survival of patients affected by chronic illnesses. Among them, left ventricular assist device (LVAD) has represented a viable solution for patients with advanced heart failure (HF). Even though the LVAD prolongs life expectancy, patients’ vulnerability generally increases during follow up and patients’ request for the device withdrawal might occur. Such a request raises some ethical concerns in that it directly hastens the patient’s death. Hence, in order to assess the ethical acceptability of LVAD withdrawal, we analyse and examine an ethical argument, widely adopted in the literature, that we call the “descriptive approach”, which consists in giving a definition of life-sustaining treatment to evaluate the ethical acceptability of treatment withdrawal. Focusing attention on LVAD, we show criticisms of this perspective. Finally, we assess every patient’s request of LVAD withdrawal through a prescriptive approach, which finds its roots in the criterion of proportionality.

## Preliminary Framework

Over the last century, technological advancement has given birth to ethical concerns over the role of *téchne* in clinical practice and the emergence of ethical implications of its utilization in end-of-life issues.

Chronic illnesses, the most predominant in contemporary clinical practice (Milani and Lavie 2015), are more challenging than acute diseases because they require ongoing therapy until the very end of life. In such clinical cases, patients live in a permanent pathological state of deficiency that they must learn to live with.

Advanced heart failure represents one of the major chronic pathophysiologies against which technological advancement tries to fight. According to the American Heart Association, heart disease remains the first cause of death in United States of America (USA).

According to the National Health and Nutrition Examination Survey (NAHNES), the 2018 report of the American Heart Association (Benjamin et al. 2018) observed that 6.5 million adult Americans suffered from heart failure (HF) from 2011 to 2014; this result represented a significant increase as the same statistics, for the 2009 to 2012 period, showed that less than 6 million people—just under 5.9 million US adults—suffered from HF.

As the number of patients with HF continues to rise in contrast to that of donors available for heart transplant, and as the time spent on waiting list is unpredictable, clinical development in mechanical circulatory support (MCS) devices represents an important contribution for patients’ survival (Stewart and Givertz 2012; Burkhoff et al. 2015).

The structure of support devices has improved significantly and the new generation of left ventricular assist devices (LVADs), the most common in current clinical practice (Eisen 2019), has become less troublesome for recipients (Stewart and Givertz 2012). In short, as the Eighth Interagency Registry for Mechanically Assisted Circulatory Support (INTERMACS) annual report shows, the development in technological circulatory support has constituted an innovative alternative for patients suffering from heart failure, reducing adverse effects, improving life expectancy and patients’ overall quality of life (Kirklin et al. 2017; Mancini and Colombo 2015).

## What is a LVAD and Why This Implant Raises Ethical Concerns

Left ventricular assist devices (LVAD) are mechanical pumps that replace the pumping function of the left ventricle to push blood into the aorta and maintain an adequate blood flow. Unlike the first LVADs, the new generation LVADs are surgically implanted into the chest of the patient and connected to an external controller which functions through a power source that must be recharged electrically, either with batteries or with an outlet. Supplying the function of the left ventricle, LVADs can be adopted for the management of advanced heart failure through four different strategies: bridge to transplant (BTT), bridge to candidacy (BTC), bridge to recovery (BTR) and destination therapy (DT) (Stewart and Givertz 2012; Smith et al. 2015). Unlike the first three strategies, belonging to the category of “temporary circulatory devices”, DT LVAD implants are more challenging and harder to manage. In such circumstances, the device is a viable, long-term support solution for patients ineligible for transplant because of their physical limitations. The device represents a sort of “extraordinary” opportunity for patients exposed to the risk of imminent death; however, the period after the implant is described as a “liminal state” (Standing et al. 2017), during which recipients feel neither healthy nor critically ill (Barg et al. 2017).

Unlike the BTT strategy, even though patients in DT feel grateful for having been selected as good candidates, they generally express a lack of choice and powerlessness due to their double dependence on the machine and on their caregiver, the significant and permanent lifestyle trade-offs during the follow-up phase (Rady and Verheijde 2014) and the myriad of collateral effects, such as infections, bleeding, device malfunctions, stroke and multi-organ failure that could trouble the patient’s conditions (Kirklin et al. 2017). This explains why the worsening of LVAD patients’ quality of life may incite their eventual request for the deactivation of the device (Rady and Verheijde 2014; Rizzieri et al. 2008).

The ethical acceptability of LVAD deactivation is being challenged because of the meaning attributed to the perfusion it produces. After the implant, the continuity of blood perfusion is guaranteed by the mechanical pump. In fact, although the DT LVAD is first to “support” the left heart chamber, once implanted, it soon replaces the native function of the ventricle, maintaining an adequate blood pressure for the rest of the body. Since any cardiac device such as LVAD, pacemaker and implantable cardioverter-defibrillator (ICD) has its own function and mechanism, end-of-life trajectories vary depending on the partial or complete perfusion of the device implanted, complicating end-of-life decision making (Rady and Verheijde 2014). Thus, it is fundamental to highlight that both collateral events after the withdrawal and the length of the dying process depend on the nature of the device implanted.

In the particular case of patients with a DT LVAD implant, the native organ does not function on its own even though still has its place in the chest, to such an extent that the patient, without an LVAD, could die. The dying process lasts from 20 minutes up to 48 hours.

In this sense, we can argue that the LVAD is a full life-saving support, just like any transplanted organ is. The patient wholly depends on its activation, just as any transplanted patient depends on their new perfused heart. Therefore, the patient’s request to deactivate an LVAD raises delicate issues concerning end-of-life trajectories and decisions.

Based on these observations, it will be necessary to understand whether the withdrawal of the LVAD is deemed an act morally equated to “killing” (Marin 2017; Bramstedt and Wenger 2001; Kini and Kirkpatrick 2013).

In order to pursue this line of thought, it is essential to shift from a descriptive level to a prescriptive one. The descriptive approach consists in giving a definition to the notion of “life-saving treatment” in order to justify whether a LVAD, according to its definition, might be switched off and in what circumstances this might be conceived as an act of euthanasia.^1^

Should the internality-externality argument make deactivation ethically acceptable? In other words, should the structural composition of a medical device influence the decision to end the patient’s life? Might a normative assertion on withdrawal derive from a descriptive assertion over the structural definition of a treatment?

The analysis will shift to the normative approach, once the impasse of the “descriptive approach” is demonstrated. Reconsidering the notion of “futility” by Pellegrino, the criterion of proportionality will be explored with the goal of understanding in which circumstances it will be ethically acceptable to turn a LVAD off and how such a criterion legitimates the choice to deactivate the device when the burdens outweigh the benefits, allowing the patient die in dignity.

## The Descriptive Approach

### The Internality–Externality Dualism: The Hybrid Composition of LVAD

The first binary definition, which can be found in the literature to define a life-sustaining treatment, concerns the distinction between internal and external devices. The existing difference between the internal and the external composition of the structure of a treatment comes from the fact that a device, according to its mechanical structure, might be objectively considered as an internal or an external part of the body itself (Sulmasy 2007; Jansen 2006).

The notion of “internal” treatment indicates that a medical treatment is located internally, becoming a new integrated part of the organism, that is, an integrated part of the self much as any transplanted organ. The LVAD, in fact, is partially internal since it is surgically implanted into the chest, constituting an ancillary support for the heart. In the paper “Doctor, will you turn off my LVAD?”, Simon (2008) explains that, besides the external controller, the LVAD is “an integrated part of an independent functioning organism” (p. 14). Since it completely fulfils the perfusion of the native heart internally, its activation implies the support of the patients’ functional organism, becoming a new part of the body. Considered as internal and integral as the heart, its integration is a condition that does not allow the deactivation of the device.

On the other hand, the concept of “external treatment” states that a clinical therapy is located outside the patient’s body and that it does not belong to the self: there is no integration between the therapeutic option and the body, no effective physical interaction. The body defines its physical boundaries and treatment is conceived as an “outsider”. In other words, the externality of therapeutic treatment does not directly affect the patient’s body as a transplanted organ does. Unlike Simon’s position, Fischbach (2008) argues that since LVAD is not an internal organ, its maintenance requires an ongoing external engagement both for patients and caregivers. This implies that, in accordance with externality, the LVAD may be switched off.

The structure of a LVAD is different from other life-sustaining treatments. LVAD has a hybrid composition (Kraemer 2013) as it is partly inside the body—the mechanical pump is surgically implanted into the chest and a tube is internally connected to the left ventricle to push blood into the aorta—and partly outside the patient’s body—it is connected via cord to an external computer that has to be recharged electrically. Because the LVAD is neither a completely external treatment (e.g., mechanical ventilation), nor a completely internal one (e.g., pacemakers), the argument of the “internal/external” dualism would not be consistent and sufficient to justify the acceptability of LVAD deactivation: its hybrid composition places the device in an “in-between” space where the boundaries between “internality” and “externality” are undefined. The patient, while looking in the mirror, sees the controller and the batteries that allow the pump to function. On the one hand, this sort of “in-betweenness” and limbo does not make the patient feel free and independent from the external source. On the other hand, the mechanical pump implanted into the chest is what makes the patient feel still alive, despite its partial externality. The feeling of being always in a “liminal space” (Barg et al. 2017) does not always help the patient understand what the LVAD is for her/him; that is, its meaning in the construction of her/his personal identity after the implant.

At present, bioengineering has not succeeded in developing either a totally internal LVAD, or a completely internal Total Artificial Heart (TAH); that is probably due to the presence of both a controller and a power supply (i.e., batteries), which are too large to be implanted in the body. But if one day the total implantation were possible, thereby overcoming the complexities of the hybrid composition of MCS, should we equate LVAD withdrawal with an injection of KCI (potassium chloride) for the discontinuation of the transplanted heart on the base of the “internality thesis”? Should the two acts be considered acts of euthanasia?

Following the Sulmasy’s definition (Sulmasy 2007, p. 70), “killing” constitutes “an act in which an agent performs an action that creates a new, nontherapeutic, lethal, pathophysiological state in a human being with the intention of causing that human being’s death”. It is possible to notice that according to such a notion, as the author continues, there would be no difference *in the result* between the deactivation of an internal and of an external treatment, e.g., between an injection of KCI to stop a transplanted heart and an internal device deactivation: both result in the patient’s death. However, if the former produces an act which introduces directly a new lethal pathophysiology accelerating the process of death, the latter allows the cardiac disease affecting the patient to run its course until the end.

### The Second Dualism: The Distinction Between “Replacement” and “Substitute” Treatments

Unlike the “internality vs. externality” thesis, the present differentiation focuses more on the functions that a treatment fulfils, rather than on its mechanical structure (internal or external).

Contrary to acute diseases that can be cured with “regulatory treatment”, helping the body to re-establish its haemostatic equilibrium regulating a particular function of the organism through external or internal administration, the so called “constitutive therapies” provide a function that the body cannot fulfil by itself (Rady and Verheijde 2014). Two kinds of life sustaining treatments belong to such a category: the replacement treatment and the substitute treatment. The former states a medical therapy completely replaces an organ function, e.g., a transplanted heart. Put differently and as Sulmasy explains (Sulmasy 2007), replacement therapy provides a function that was lost in the organism because of the progress of the disease. Rady and Verheijde (2014, p. 6) list the main elements of replacement therapy, such as “responsiveness to changes in the organism, properties as growth and self-repair, independence from external electrical source, independence from the external control of an expert, immunological compatibility and physical integration into the patient’s body.”

According to this definition, replacement treatment, such as a cardiac transplant, is totally integrated into how the organism functions: it responds to its needs and is embodied in its metabolic changes. The act of withdrawing a replacement treatment directly interrupts the organic integration of the treatment in the organism, producing a new lethal pathophysiology, thus harming the patient and hastening the process of death (Sulmasy 2007).

The withdrawal would correspond to an intrusive action, compromising the patient’s well-being and the clinical effectiveness of the treatment itself.

On the other hand, Rady and Verheijde define substitute treatment, e.g., a cardiac device, in the following terms: it is a permanent replacement of a native physiological body function, with continuous control of electrical functions of the heart; it is integrated in the patient’s body and immunological system, responding to all its changing demands; moreover, if respondent to the body changes, these devices are dependent on an electrical source and on the physician’s judgment. In other words, by substituting for the native function of the heart, cardiac device deactivation determines the gradual cessation of circulation, respiration and consciousness.

In the paper by Rady and Verheijde (2014), it is essential to underline that as opposed to replacement therapy, substitute treatment is completely dependent on an electronic source and on the judgment of an expert. During the follow up with a LVAD, batteries have to be changed or recharged every 8 to 12 hours by caregivers and the controller has to be monitored both by patients, caregivers and MCS clinicians of the LVAD centre, in the aim to verify the occurrence of unexpected abnormalities, device malfunctions or collateral effects. Whenever the patient expresses sickness in handling the everyday difficulties of the implant, the request to deactivate the device might arise.

Even though the LVAD becomes the condition *sine qua non* the patient would die, it is evident that mechanical circulatory supports do not cure patients from the underlining heart diseases (Bramstedt 2003a,b). On the contrary, the mechanical circulatory devices only support the pathological state replacing the pumping function of the native heart. That means that the pathological state does not disappear with the treatment itself because it does not constitute a complete cure.

Going back to Sulmasy’s definition of “killing” (Sulmasy 1998), the discontinuation of a “replacement treatment”, which has become an integrated biological part of the body itself, necessarily introduces a new lethal pathophysiology, leading to the patient’s death. Whereas, “allowing to die” indicates the act of removing a treatment for the pre-existing disorder or the renunciation to start a therapy.

With regard to the withdrawal of substitute treatments, consider two different scenarios that might concern a DT LVAD implant:

- The first has to do with the interruption of a LVAD whose burdens and collateral effects become so significant during the follow up phase that the patient is not able to handle everyday life with the device. On the one hand, treatment deactivation might produce some clinical consequences, which the palliative care team is called to deal with, on the other hand, once the LVAD is switched off, the patient’s death is principally determined by the previous pathological state and the several burdens perceived during the therapy. In other words, when the treatment becomes ineffective and disproportionate to the benefits, deactivation allows the death process to follow its natural course.

- The second scenario concerns the case of a patient with a DT LVAD, who is not significantly suffering from the collateral effects produced by the implant and who is not at risk of imminent death. Should the patient’s request to switch the LVAD off be respected? Are there any other factors to take into consideration for the clinicians to accept such a request?

This second scenario challenges the possibility to withdraw the implant in that collateral effects do not particularly affect the patient and the clinical conditions are stable. In this kind of scenario, it is fundamental to realize an assessment of all the factors that could play a role in the patient’s choice, e.g., depressive symptoms, psychiatric diseases, feelings and changing perceptions about the DT LVAD, a good relation with a caregiver who takes care of the patient after hospitalisations. Therefore, it is fundamental to identify the circumstances in which the LVAD is effectively disproportionate for the patients’ conditions from the situations in which the request is expressed when the pathological state is not progressing, and the patient is not facing death. In the second case, if the request is accepted with no opposition or discussion between clinicians, patients, and families, the interruption of the therapy risks being conceived as a patient-assisted-death (PAD) (Rady and Verheijde 2014). The second scenario evidently leads us to consider the fact that the patient’s stable condition is guaranteed by LVAD perfusion. This argument shows that the definition of substitute treatments is not always fit to ethically justify every deactivation of the support device; on the contrary, it will be fundamental to analyse circumstances and variables on a case-by-case basis.

In fact, heart transplant differs from a mechanical device primarily because of its “natural biofixture” (Rizzieri 2008; Paola and Walker 2000), replacing the pathologic native heart with a new healthy organ. A LVAD does not physically replace the native heart but supports it by pumping blood and substituting its lost pathologic function. Ultimately a transplant and a LVAD deliver the same result, maintaining the heart working and preserving life.

What emerges from this parallelism is that even though in both cases life is literally sustained by the medical treatment, the former is closer to the “naturality” of the native heart, while the second supports the hemodynamic functions of the native heart “from the outside”. This kind of distinction may be equivocal in that it suggests that transplants completely cure the patient. On the contrary, as Bramstedt suggests,

heart transplants (allografts) are not seen as curing heart disease but a therapeutic intervention in the larger picture of the health of the cardiovascular system. Heart disease processes can still occur after allograft transplantation. Refusing re-transplantation or other interventions can be viewed as allowing the underlying cardiac disease process to take its course to death (Bramstedt 2004, p. 427).

Bramstedt’s assertion is partly sharable because heart disease may also occur after the transplant and is not limited to the heart but involves the entire cardiovascular system. Moreover, rejection episodes may occur because of other collateral factors, directly linked to the immunosuppressant therapy.

In heart transplantation, the assessment of proportionality is realized before the transplant, during the patients’ selection for transplantation (Lampert and Ramani 2017). Even though the patient may be affected by rejection episodes, personality disorders, depressive symptoms, distortion of the self and psychological difficulties in managing the changes of identity, the health care team aims at preventing organ rejection and relapse during the follow up (Kraemer 2013). Literature illustrates the difficulty patients face in accepting a new organ, that is, an outsider; however, this feeling of strangeness does not generally lead to the request of organ withdrawal (Wilhelm 2013). This act is not even conceived since it corresponds to a clinically invasive surgery leading directly the patient’s death. Such an act, in fact, is against good clinical practice, harming the patient and involving the patient’s death intentionally. As Sulmasy argues (1998, 2007), respecting the patient’s request to withdraw a replacement treatment, like a transplanted heart, is morally illicit and constitutes an act of killing because, unsupported by any clinical conditions, it would produce a new and lethal pathophysiology. Instead of asking to have the heart removed, the patient might let himself/herself die, for example, by ending immunosuppressant therapy.

Unlike heart transplantation, however, patients’ conditions with LVAD may worsen not only because of the underlying cardiac disease but also because of the unforeseeable collateral events, also related to the cardiovascular system, which may have an important impact on patients’ end-of-life trajectories, deteriorating their course of life. Thus, like in the first scenario, the deterioration of the clinical picture may influence patients’ values and preferences in their end-of-life, leading them to request the LVAD to be switched off.

Is “artificiality” the criterion for the ethical acceptability of LVAD withdrawal?

In order to override the *impasse* of the descriptive level, the distinction between “natural” and “artificial” may represent the binary pair at the base of the incoherence of the two above-mentioned dualisms (internal vs. external devices; replacement vs. substitute treatments). In fact, even though some studies use the notion of “biofixture” in order to state a sort of belonging of a device integrated in the patient’s body (Paola and Walker 2000; Kramer et al. 2010; Jansen 2006; Sulmasy 2007), it is essential to re-evaluate the concept of “nature” and *“*artificial” and the role it may have in the decision to deactivate a treatment.

The notion of “nature” has progressively changed its meaning in relation to the empowerment of technical progress. If we took the Aristotelian teleological conception of “nature”^2^ as the principle with its own movement, while the “artificial” as the product of an external force that interrupts the course of nature and its internal organization, it would be legitimate to assert that every outcome of the medical *téchne* is both a distortion of nature itself—a deviation from the natural course of human life, incapable of overriding illnesses by itself—and the effort to preserve human existence.

The empowerment of *téchne* has completely changed the teleological perspective of nature, as a normative process of self-handling. The progress of bioengineering and the technological imperative have, therefore, modified the conception over the notion of human nature and the management of human lives in the healthcare setting.

It is fundamental here to make a distinction introduced by Robert Spaemann (2002).

For the German philosopher, there exists a substantial difference between *being* a nature and *having* a nature: the former indicates the ontological structure of a particular reality, the internal principle determining the definition of “being human”; in the particular case of the human being, as Kant suggests (Kant 2011) this principle is dignity, which distinguishes human beings from objects that have a price; the latter, having a nature, comparable to the Kantian “material” definition of nature, represents everything that can be an object of experience through reason, i.e., the totality of phenomena. Therefore, it is because we *are* a particular nature, e.g., humanity, that we all *have* a nature, e.g., reason. Thus, as Spaeman argues, nature should be defined as both the condition and the content of human freedom, which accomplishes itself through reason.

*Having* a nature, as an expression of human reasoning, has progressively changed its meaning, becoming a disposition for the *domination* of nature through human reasoning itself: far from indicating the innate tension for the contemplation of truth, as Plato suggests in his philosophical dialogues, human reasoning has turned into instrumental rationality, as well-explained by Adorno and Horkheimer (2002).

This technical rationality has affected the empowerment of the medical settings: the human body is a machine to be controlled in order to prevent the progression of illnesses.

It is evident that, on the one hand, the technological imperative answers the need to cure patients and, on the other hand, bioethics progressively focuses the attention on the importance of caring for them. Treatment withdrawal is an evident manifestation of clinicians’ caring approach whenever no cure might accomplish the objective of restoring health.

Mechanical devices and organ transplantations are two examples of such a technical intervention in medicine. Neither the former nor the latter can be considered a natural process of re-establishing health*:* both heart transplants and cardiac devices are the result of the empowerment of medical science; that is, each is a direct manifestation of the interruption of the natural course of life, whenever it is affected by illness, in order to preserve it.

Moreover, the notion of replacement treatment, e.g., cardiac transplant, refers to “the responsiveness of a treatment to changes in the organism” (Rady and Verheijde 2014): in transplantations, responsiveness to the organism is also determined by the immunosuppressive therapy, which is a fundamental condition for the patient’s outcome in the follow up. This claim aims at emphasizing that cardiac transplant depends on the immunosuppressive therapy as well as on a healthy diet and appropriate conduct, even if it replaces the native organ. This means that “external” factors are fundamental to the patient’s outcome during follow up.

The new heart physically replaces the pathological one, but it necessarily depends on other external but essential conditions. Such reasoning demonstrates that just as cardiac transplant is not a complete replacement, because as it depends on other variables, the definition of “substitute treatment” is also adequate to ethically accept the withdrawal of a LVAD because the device is not a complete “substitution”; therefore, the deactivation of the device has to be legitimated by a normative statement considering the factors having an impact on end-of-life cases.

## The Criterion of Proportionality: How does it Provide Acceptability to LVAD Withdrawal?

Far from wondering whether a categorization of the LVAD, by definition, may be essential to ethically explain the legitimacy of deactivation, it may be more appropriate to reflect over the conditions implying the patient’s potential request of withdrawal and in what circumstances physicians may accept such conditions, agreeing with the patient’s wish. In order to achieve this objective, it will be necessary to reflect upon the criterion of proportionality, considered as the *rationale* justifying the physician’s decision to interrupt treatment, and adhering to the patient’s will. Moreover, the explanation of this criterion will help us take a well-argued position in the euthanasia debate, explaining why and under which conditions LVAD withdrawal is not an act of euthanasia.

The notion of proportionality is not monolithic; on the contrary, it is the result of the assessment of a diversity of factors transcending the patient’s clinical conditions, but closely linked to them.

To better understand the use of this concept in clinical practice, the term *medical futility* coined by Pellegrino may be particularly relevant (Pellegrino 2000a,b, 2005).

We consider the term “futility”, as Pellegrino suggests, is a “means for prudential clinical judgment” and is particularly useful to distinguish ordinary from extraordinary treatments in the clinical practice. If we take prudence to mean “practical wisdom”—the Greek *phronesis*—described in the sixth book of Aristotle’s *Nicomachean Ethics,* which is the human disposition to discern the good in specific and contextual situations, and if we translate, as Pellegrino suggests, “practical wisdom” into “the prudential guide to moral assessment to the good of the patient and to the moral permissiveness of withholding or withdrawing particular treatments in seriously ill or dying patients” (Pellegrino 2000b), it is possible to claim that futility expresses a judgment that finds its roots in the complexities of particular dilemmatic circumstances on the patient’s good in end-of-life circumstances.

In his genealogy of the notion of “futility”, Pellegrino (Pellegrino 2000b) distinguishes the denotative conception of the term from its connotative value. If the former is used to define something that is inadequate for the expected goal, e.g., a particular treatment is not effective for the restoration of health, the latter is enriched by all the imaginative associations the term might refer to—in our specific case, a futile treatment might suggest the idea of the abandonment of the patient. Considering the presence of a variety of declinations of the term in daily use, Pellegrino is considered the father of the notion of futility in that he reviewed the concept and underlined the difference between its classical use and the need for an renewed meaning to attribute to such a notion.

In fact, past tradition translated futility from a clinical perspective. Re-proposing the difference between ordinary and extraordinary treatments, the notion of futility was generally used to indicate a therapy where pains and costs were more significant than benefits produced. In these circumstances, the treatment was “extraordinary” in that it prolonged human life and pains without an aim. Therefore, as Pope Pio XII suggested in a speech in 1957, in order to respect the person’s dignity, the withdrawal of any extraordinary treatment may be considered licit. Even though at that time futility still maintained its technical and clinical meaning, the development of the movements for the recognition of the patient’s rights and the proclamation of the principle of autonomy as the pillar of bioethics and clinical practice have progressively determined a new and different declination of the concept of futility.

In 1980, the Sacred Congregation for the Doctrine of the Faith published a Declaration on Euthanasia where the notions of “extraordinary” and “ordinary” treatments were replaced by “proportionate” and “disproportionate” means. Proportionality was thus conceived as a judgment realized on the basis of “the type of treatment to be used, its degree of complexity or risk, its cost and the possibilities of using it, […] comparing these elements with the result that can be expected, taking into account the state of the sick person and his or her physical resources” (Sacred Congregation for the Doctrine of the Faith 1980).

In the light of these observations, the judgment of proportionality might be translated into Aristotelian practical wisdom, or “prudential guide” by Pellegrino at the base of his notion of futility.

The Vatican definition of proportionality highlights the eclectic composition of this judgment.

Thus, it is essential to recognize the importance of the technical role of the physician and his/her strict clinical risk–benefit ratio in the evaluation of any treatment effectiveness. However, this ratio alone underestimates the value and the function of the patient-physician relationship in identifying a good choice, especially in critical circumstances, such as the activation or the withdrawal of a LVAD. It is through the interaction between two moral agents, the patient and the physician, their life stories and autobiographical experiences that any clinical decision might be taken towards a “good decision”.

Even though Pellegrino did not directly use the notion of “proportionality”, but rather that of “futility”, his explanation efficiently clarifies the factors that must be taken into account whenever considering the appropriateness of a therapeutic option; these variables belong both to the clinical domain and to the patient’s world of values, goals and context: the first is effectiveness, the second deals with benefits, and the third with burdens (Pellegrino 2005; Pellegrino 2000a,b).

Effectiveness is related to the clinical sphere and consists of the physician’s ability to alter, through techno-medical expertise, the disease process: a treatment is effective if it clinically fulfils the desired end, that is, the patient’s well-being. Benefit is more related to a subjective determination of what is good for the patient; this evaluation is realized by the patient, if competent, or by a valid surrogate. Unlike the first element, benefit is assessed based on patient’s values and goals in undergoing a treatment, that is, the patient’s possibility to pursue his or her life objectives. The third factor regards burdens, which are evaluated on the bases of physical, social, existential, and economic costs. Not only are objective costs decisive for such an evaluation; both caregivers’ and patients’ concerns are involved in order to assess if a treatment is futile.

It is essential to underline that effectiveness, benefits, and burdens must be equally considered for the assessment of the treatment's proportionality. If the overall evaluation of these three factors does not respect the patient’s good, the treatment must be considered futile.

The same observation is realized by Fleming (2005). In his paper, futility, far from being conceived as an objective term based on prognosis, life expectations, and assessment of clinical improvements, is found as an integration of variables, which join both the clinical sphere of effectiveness and the patient’s world of values, preferences and burdens. As Flaming explains, the content of “futility” is “moral”, more than merely clinical, in that its aim is to valorise the intrinsic essence of human beings, that is, dignity.

Whenever a treatment becomes burdensome because its costs (not merely physical, but especially psychological, relational, and existential) overweigh its benefits, its application might prolong quantitative human mortal life, sustaining the biological functioning of an organism but the patient’s body is not experiencing nor living. The dimension of “living” is determined by the interaction of the double dimension of the *body*, that of the “to have” and that of the “to be”^3^ (Merleau-Ponty 2012): the fact of *having* an exterior body with functions that still respond to the organism through a medical treatment might meet the “to be”, the dimension implying the interaction of the deeper trace of subjectivity. Therefore, if the body is conceived not only as what the individual has, but also the element through which the individual is and expresses himself/herself, we argue that any treatment, beyond the quantitative prolongation of human life, has to reflect the inner desires of subjectivities, their perception of burdens and networks outside the hospital walls.

On the basis of this final observation, we argue that the notion of futility by Pellegrino is particularly relevant in that it represents the “prudential guide” (Pellegrino 2000b) for the expression of an ethical judgment that, in end-of-life situations, helps the stakeholders to make a decision for the good of patients, even when this kind of decision implies withdrawing treatment.

The choice to shift from the notion of futility to that of proportionality is due to a “methodological” reason. As Pellegrino suggests too, the two concepts are synonyms.

However, according to our perspective, if the former refers to the process of guiding the decision to make, the latter evokes the criterion based on which the exploration is realized.

The criterion of proportionality is fundamental when facing ethical dilemmas for the deactivation of life-sustaining treatments, like LVAD.

Many collateral effects that hamper the follow up and prevent the patient from leading a “normal life”, bear a weight over their desire to switch a LVAD off (Arnold et al. 2016). The device might improve qualitatively the overall expectations of survival, but that does not constitute a guarantee. Stroke, infections, multi-organ failure, device malfunctions are all unpredictable collateral episodes that might aggravate the patient’s outcome during follow up, while prolonging life and sufferings (Rady and Verheijde 2014). An overall progressive deterioration of clinical health conditions during the follow-up challenges the patient's psychological stability. It also makes their personal life-goals uncertain, decreasing the patient’s desire to go on with treatment; in other words, the request for withdrawal meets clinical approval in that treatment is clinically ineffective and unnecessary for the patient’s physical and personal wellbeing, but especially disproportionate in respect to the complexity of the patient's suffering. Deactivation of the device corresponds to an extreme gesture of care and humanity by the physicians, while also respecting the patient’s dignity, that is, the intrinsic value of the person, a value that the preservation of life might lose when suffering and pain become burdensome (Mueller et al. 2010). Put differently, treatment withdrawal represents the practical condition for death to happen. Its aim is not death itself, but to allow nature to follow its own course (Sulmasy 1998), relieving the patient from suffering and excessive pain. This explains why LVAD withdrawal does not correspond to an act of killing. On the contrary, it is the evident recognition that the LVAD no longer fulfils its function.

In LVAD therapy, as we have already stated, clinical conditions have an important impact, even if not an exclusive one, on the patient’s outcome during follow up, as clinical factors may worsen the overall conditions of the patient (physical and psychological), harming them and altering the proportionality of the treatment itself during the follow up phase both in the hospital and at home.

Differently from other kinds of life-sustaining treatment, like ICD or pacemakers, the boundaries defined by LVAD functions are not clear. If at first it supports the pumping function of the left ventricle, it progressively becomes the *only* condition for the cardiac activity. That is why the distinction between replacement and substitute treatments by Sulmasy or Rady and Verheijde is not applicable for the LVAD activation. Sulmasy (2007), after defining “replacement” therapies as “part of the restored physiology of the patient” and after pointing out that the more a technological intervention is conceived as a “replacement” becoming “part of the restored physiology of the patient, “an integrated part of the organism”, the more the withdrawal represents a “killing”, he explains that there exists no “absolute standard” for the categorization of substitute and replacement treatments. And the LVAD belongs to a “grey zone” in that it is “between the two”: the discontinuation of LVAD leads the patient’s heart failure to its natural course until death and to the progressive deterioration of the other vital functions of the organism.

In the light of this observation, the request for LVAD withdrawal might not be ethically justified by the argument proposed by Sulmasy; it is more appropriate, in end-of-life situations with a DT LVAD, to assess the proportionality of the implant, considering treatment effectiveness, patient’s wishes and preferences, and the overall burdens making the therapy futile at a certain point of follow-up (McGee 2011).

Another important aspect to take into consideration when treating the ethical issue of treatment withdrawal has to do with the implications of the dying process. Since the waiting time for death after the deactivation of the device varies from 20 minutes up to 48 hours and the arrest of circulation determines the progressive deterioration of vital functions, it is fundamental to guarantee the patients a continuity of care supported by the intervention of a palliative care team (Wordingham et al. 2017). For this reason, the International Guidelines for MCS published in 2013 (Feldman et al. 2013), dedicate an entire part to advance care planning, palliative care collaboration, and the option of deactivation. Evaluation of the appropriateness of deactivation must consider the possibility of several complications that might occur during the process leading to death; therefore, the healthcare team must ensure support so that patients will live the phenomenon of death as peacefully as possible.

As we suggest, proportionality is a judgment expressed by physicians taking into consideration treatment effectiveness, its benefits and burdens. It is a global judgment realized for the good of the patient. When a LVAD is no longer effective because clinical adverse events and burdens are more than benefits, that is, when the treatment is no longer appropriate for the improvement of the patient’s cardiac conditions and overall quality of life, the deactivation of the device may be considered as part of the process of care itself, letting the patient die whenever sufferings and pains become intolerable and excessive (Guidry-Grimes and Sederstrom 2015). In this sense, we might state that taking care of the patient also means stopping the treatment and letting death take its natural course.

Conversely, the withdrawal of a transplanted heart or the induction of its arrest through KCI injection might not be equivalent to LVAD withdrawal for two main reasons. First, the formers are considered acts that harm the patient and introduce a lethal pathophysiological state that interrupts the integrity of the organism. The technical knowledge of a physician is needed to complete the withdrawal of a transplanted heart or to furnish the KCI injection; in other words, the involvement of clinicians—directly in withdrawal, and indirectly in offering the injection—would contribute to realize an act against good clinical practice. In the first case, the physician would commit an act of euthanasia, while, in the second, he would set the context for patient assisted suicide (PAS) would be constituted. The second aspect deals with the assessment of appropriateness of the right candidates for transplantation during the selection phase. We have already argued that with LVAD patients, the criterion of proportionality depends on a timeline that influences the appropriateness of the therapy due to clinical outcome, preferences, and burdens experienced during the follow up. At the end-of-life, when burdens are unbearable, futility makes the deactivation of the cardiac device ethically acceptable. Deactivation might be read as the accompaniment and the support of patient until the last step of life. Meanwhile, heart transplantation being a surgery that recreates an organic integration of the new organ in the organism, demands an act that harms the body, leading to death. There are unquestionably patients who experience follow up after transplantation with difficulties and relapses: in these complex circumstances, it is sometimes possible to propose another transplant, or, if that is not possible, physicians might continue to support and take care of the transplanted patients during the process of dying. In this phase, no difference exists between transplanted patients and LVAD ones, supported by palliative care specialists. However, there is no possibility for the patient to ask physicians to stop their heart. This would be an act of euthanasia.

To sum it up, a fundamental element must be highlighted to assess proportionality. Patients’s preferences don’t exist on their own; other factors play a role in such an evaluation. This means that preferences must be evaluated considering clinical effectiveness too. The patient’s values and burdens must be read in the light of their overall clinical picture. For this reason, LVAD’s unpredictable collateral effects may worsen clinical conditions, causing an important impact on the patient’s preferences, increasing excessive and intolerable pains. These unpredictable adverse events procured by the device may influence the way in which the patient experiences the LVAD, that is, the way benefits and burdens are perceived. This suggests that the *biographical lifeline* of a patient with a DT LVAD, a lifeline characterized by changing burdens and benefits, might constantly be read in relation to their clinical picture—the clinical effectiveness of a treatment, since adverse clinical events are the major cause of the deterioration of quality of life. Thus, proportionality is a criterion that must be constantly embodied in the light of the transformations of both clinical conditions and the impact of these factors in the subjectivity of the suffering patient.

A patient’s identity is not monolithic; it is not restricted to the mere fact of “being a patient”. The LVAD produces collateral effects that could increase the sense of helplessness and vulnerability; however, it is fundamental for the patient to be accompanied by the caregiver and the clinicians in a path making him/her aware, whenever possible, of end-of-life alternatives in accordance to new wishes, values, and beliefs. LVAD deactivation may reflect one of these desires.

As the so-called “patient’s preferences” are not separated from the illness affecting the patient, in the same way, clinical complications of the follow up might have an impact over end-of-life decisions. This explains why in critical end-of-life circumstances, if the patient expresses the desire to interrupt the LVAD therapy, clinicians are allowed to support such a decision, accompanying the patient to die.

## Conclusion

The empowerment of medical technology leads us to reflect on its boundaries over human finitude. Mechanical Circulatory Supports are considered life-sustaining therapies for patients with advanced heart failure. The LVAD is a sustaining treatment replacing left ventricle function. Stopping it would mean hastening the process of death. Like other life-saving technologies, e.g., mechanical ventilation, there are circumstances in which the treatment is no longer appropriate for the patient’s good.

Difficulties in living a “new normal” with a LVAD and collateral effects after the implant are well-known.

The present analysis highlights that a descriptive definition of LVAD is not enough to analyse the euthanasia debate. We overcome the dualistic nature of the various arguments, showing their ineffectiveness to address the grey scale characterizing every human existence and dilemmatic clinical cases.

Thus, we suggest that the criterion of proportionality might constitute the normative dimension to comprehend the reasons for LVAD deactivation and its ethical *rationale*. It is fundamental to underline that the follow up of patients with a LVAD may worsen not only by underlying cardiac disease, but also by unforeseeable clinical adverse events produced by the device implant. Proportionality helps to detect those variables—clinical, subjective, contextual—influencing every stakeholder's *life story* and defining every clinical situation in order to have a better understanding of every one of them and their impact on the final decision. In this *shared direction*, LVAD withdrawal will represent an extreme gesture of care in order to respect the patient’s free decision-making process.

## Footnotes

1 The starting point of our analysis follows the line of the work realized by Felicitas Kraemer, over the “ontological approach” (Kraemer 2013) for the justification of any treatment withdrawal. For the author, the term “ontology” is used to define a LVAD, that is, to answer the question “What is a LVAD?”. Since the concept of “ontology”— which corresponds to the philosophical discipline studying the metaphysical structure of the “to be”—might be misleading whenever facing issues related to clinical ethics, a “descriptive approach” may be more appropriate.

2 The adjective “teleological” derives from the two Greek notions of “telos” (end, purpose) and “logos” (reasons, explanation, argument) and literally indicates the “explanation over the end”. Aristotle is one of the first pioneers of natural teleology which consists in asserting that every change has its own principle and end in nature itself.

3 The philosophical distinction between “having a body” and “being a body” was first introduced by Husserl and his phenomenology and then revisited by Merleau Ponty’s Philosophy of Chair.

## References

[CR1] Adorno, T. W., & Horkheimer, M. (2002). *Dialectic of enlightenment*. Trans. Edmund Jephcott. Palo Alto: Stanford University Press.

[CR2] Aristotle. (2012). *Physics*. C. D. C. Reeve (trans.). Cambridge: Hackett Publishing Company.

[CR3] Arnold SV, Jones PJ, Allen LA, Cohen DJ, Fendler TJ, Holtz JE (2016). Frequency of poor outcome (death or poor quality of life) death or poor quality of life after left ventricular assist device for destination therapy: Results from the INTERMACS Registry. Circulation Heart Failure.

[CR4] Barg FK, Kellom K, Ziv T, Hull SC, Suhail-Sindhu S, Kirkpatrick JN (2017). LVAD-DT: Culture of rescue and liminal experience in the treatment of heart failure. The American Journal of Bioethics.

[CR5] Benjamin EJ, Virani SS, Callaway CW, Chamberlain AM, Chang AR, Cheng S (2018). Heart disease and stroke statistics—2018 update: A report from the American Heart Association. Circulation.

[CR6] Bramstedt KA (2003). Contemplating total artificial Heart inactivation in cases of futility. Death Studies.

[CR7] Bramstedt KA (2003). Replying to Veatch's concerns: Special moral problems with total artificial heart inactivation. Death Studies.

[CR9] Bramstedt KA (2004). Elective inactivation of total artificial heart technology in non-futile situations: Inpatients, outpatients and research participants. Death Studies.

[CR8] Bramstedt KA, Wenger NS (2001). When withdrawal of life-sustaining care does more than allow death to take its course: The dilemma of left ventricular assist devices. Journal of Heart and Lung Transplantation.

[CR10] Burkhoff D, Sayer G, Doshi D, Uriel N (2015). Hemodynamics of mechanical circulatory support. Journal of the American College of the Cardiology.

[CR11] Eisen HJ (2019). Left ventricular assist devices (LVADS): History, clinical application and complications. Korean Circulation Journal.

[CR12] Feldman D (2013). The 2013 International Society for Heart and Lung Transplantation Guidelines for mechanical circulatory support: Executive summary. The Journal of Heart and Lung Transplantation.

[CR13] Fischbach RL (2008). Case study. "Doctor, will you turn off my LVAD?" Commentary. Hasting Center Report.

[CR14] Fleming DA (2005). utility: Revisiting a concept of shared moral judgment. HEC Forum.

[CR15] Guidry-Grimes L, Sederstrom N (2015). Expectation and suffering with LVAD deactivation. The American Journal of Bioethics.

[CR16] Jansen LA (2006). Hastening death and the boundaries of the self. Bioethics.

[CR17] Kant, E. (2011). *Groundwork of the metaphysics of morals*: *A German-English edition*, M. Gregor and J. Timmermann (ed. and trans.). Cambridge: Cambridge University Press.

[CR100] Kramer DB, Kesselheim AS, Brock DW, Maisel WH (2010). Ethical and legal views of physicians regarding deactivation of cardiac implantable electrical devices: A quantitative assessment. Heart Rythm Case Reports.

[CR18] Kini V, Kirkpatrick JN (2013). Patient's desire for termination of destination LVAD therapy should be respected. Journal of Cardiothoracic and Vascular Anesthesia.

[CR19] Kirklin JK, Pagani FD, Kormos RL, Stevenson LW, Blume ED, Myers SL (2017). Eighth Annual INTERMACS report: Special focus on framing the impact of adverse events. Journal of Heart and Lung Transplantation.

[CR20] Kraemer F (2013). Ontology or phenomenology? How the LVAD challenges the euthanasia debate. Bioethics.

[CR21] Lampert BC, Ramani R, Subramaniam K, Sakai T (2017). Heart transplant patient selection and preparation. Anesthesia and perioperative care for organ transplantation.

[CR22] Mancini D, Colombo PC (2015). Left ventricular assist device. A rapidly evolving alternative to transplant. Journal of the American College of Cardiology.

[CR23] Marin F (2017). Il fine vita e l’attribuzione di responsabilità morale / The end of life and the ascription of responsibility. Medicina E Morale.

[CR24] McGee A (2011). Me and my body: The relevance of the distinction for the difference between withdrawing life support and euthanasia. Journal of Law, Medicine & Ethics.

[CR25] Merleau-Ponty, M. (2012). *Philosophy of perception*, D. A. Landes (trans.). New York: Routledge.

[CR103] Milani RV, Lavie CJ (2015). Health care 2020: Reengineering health care delivery to combat chronic disease. The American Journal of Medicine,.

[CR26] Mueller PS, Swetz KM, Freeman MR, Carter KA, Crowley ME, Anderson SC, J (2010). Ethical analysis of withdrawing ventricular assist device support. Mayo Clinic Proceedings.

[CR27] Paola FA, Walker RM (2000). Deactivating the implantable cardioverterdefibrillator: A biofixture analysis. Southern Medical Journal.

[CR28] Pellegrino ED (2000). Decisions to withdraw life-sustaining treatment: A moral algorithm. JAMA.

[CR30] Pellegrino, E. D. (2000b). Decision at the end of life: The use and abuse of the concept of futility. In *The Dignity of the Dying Person. Proceedings of fifth assembly of the pontificial academy for life* (pp. 85–110). Pontificia Accademia Pro Vita: Città del Vaticano.

[CR29] Pellegrino ED (2005). Futility in medical decisions: the word and the concept. HEC Forum.

[CR102] Pope Pius XII. (1957). En reponse a trois questions de morale medicale sur la reanimation. https://w2.vatican.va/content/piusxii/fr/speeches/1957/documents/hf_p-xii_spe_19571124_rianimazione.html. Available: 1 April 2020.

[CR31] Rady MY, Verheijde LJ (2014). Ethical challenges with deactivation of durable mechanical circulatory support at the end of life: Left ventricular assist devices and total artificial hearts. Journal of Intensive Care Medicine.

[CR32] Rizzieri AG, Verheijde JL, Rady MJ, McGregor JL (2008). Ethical challenges with the left ventricular assist device as a destination therapy. Philosophy, Ethics, and Humanities in Medicine.

[CR101] Sacred Congregation for the Doctrine of the Faith. (1980). *Declaration on Euthanasia*. http://www.vatican.va/roman_curia/congregations/cfaith/documents/rc_con_cfaith_doc_19800505_euthanasia_en.html. Accessed 1 Apr 2020.

[CR33] Simon J (2008). Case study. "Doctor, will you turn off my LVAD?" Commentary.. Hasting Center Report.

[CR34] Smith LA, Yarboro LT, Kennedy JLW (2015). Left ventricular assist device implantation strategies and outcomes. Journal of Thoracic Disease..

[CR35] Spaemann. (2012). *Che cos’è il naturale? Natura, Persona, agire morale,* Rosenberg & Sellier, EAN: 9788878851566.

[CR36] Standing HC, Rapley T, MacGowan GA, Exley C (2017). ‘Being’ a ventricular assist device recipient: A liminal existence. Social Science and Medicine.

[CR37] Stewart GC, Givertz MM (2012). Mechanical circulatory support for advanced heart failure. Circulation.

[CR38] Sulmasy DP (1998). Killing and allowing to die: Another look. The Journal of Law, Medicine & Ethics.

[CR39] Sulmasy DP (2007). Within you/without you: Biotechnology, ontology, and ethics. Journal of General Internal Medicine.

[CR40] Wilhelm MJ (2015). Long-term outcome following heart transplantation: Current perspective. Journal of Thoracic Disease.

[CR41] Wordingham SE, McIlvennan CK, Fendler TJ, Behnken AL, Dunlay SM, Kirkpatrick JN, Swetz KM (2017). Palliative care clinicians caring for patients before and after continuous flow-left ventricular assist device. Journal of Pain and Symptom Management.

